# Cause-specific hazard Cox models with partly interval censoring – Penalized likelihood estimation using Gaussian quadrature

**DOI:** 10.1177/09622802241262526

**Published:** 2024-07-25

**Authors:** Joseph Descallar, Jun Ma, Houying Zhu, Stephane Heritier, Rory Wolfe

**Affiliations:** 1 School of Mathematical and Physical Sciences, 7788 Macquarie University, Australia; 2 Ingham Institute for Applied Medical Research, Liverpool, NSW, Australia; 3 South West Sydney Clinical Campuses, School of Clinical Medicine, UNSW, Liverpool, NSW, Australia; 4 School of Public Health and Preventive Medicine, 2541 Monash University, Australia

**Keywords:** Cause-specific Cox model, constrained optimization, penalized likelihood, Gaussian quadrature

## Abstract

The cause-specific hazard Cox model is widely used in analyzing competing risks survival data, and the partial likelihood method is a standard approach when survival times contain only right censoring. In practice, however, interval-censored survival times often arise, and this means the partial likelihood method is not directly applicable. Two common remedies in practice are (i) to replace each censoring interval with a single value, such as the middle point; or (ii) to redefine the event of interest, such as the time to diagnosis instead of the time to recurrence of a disease. However, the mid-point approach can cause biased parameter estimates. In this article, we develop a penalized likelihood approach to fit semi-parametric cause-specific hazard Cox models, and this method is general enough to allow left, right, and interval censoring times. Penalty functions are used to regularize the baseline hazard estimates and also to make these estimates less affected by the number and location of knots used for the estimates. We will provide asymptotic properties for the estimated parameters. A simulation study is designed to compare our method with the mid-point partial likelihood approach. We apply our method to the Aspirin in Reducing Events in the Elderly (ASPREE) study, illustrating an application of our proposed method.

## 1. Introduction

Competing risks consider the situation where there are multiple events, or multiple causes for a single event, that compete with each other. “Competing” here entails that these risks or events are mutually exclusive (e.g. Putter et al.^1^). Traditionally for competing risks data analysis, people only consider right censoring, where, if an event occurs, one will observe the event time with the corresponding risk type, and if no event has occurred, one will only observe a right censoring time without any risk type. A nice feature of a right-censored competing risks problem is that the regression coefficients of a cause-specific hazard (CSH) Cox model can be easily computed using the partial likelihood method.^2^ More specifically, for a given risk and its CSH Cox model, one can simply set the event times and right censoring times of all the other risks as right censoring times for the risk under consideration and then implement the partial likelihood method to estimate the Cox model regression coefficients; see Putter et al.^1^ and Prentice et al.^3^ However, this feature will be lost when there are interval-censored event times.

Interval-censored data can arise in competing risks. For example, when a competing risk is related with a chronic disease where its symptoms are slowly progressing, and hence the disease can only be confirmed to occur during a time interval. In this article, we consider semi-parametric CSH Cox models, where the observed event times from competing risks are partly interval-censored (e.g. Kim^4^ for partly interval censoring), which means these observed times can include competing risks event times, as well as left, right and interval censoring times. When partly interval censoring arises one may opt to maximize the full log-likelihood to estimate the model parameters. Its computation, however, is intensive^5^ and hence will not be useful without improved computational efficiency.

In this article, we will develop a Gaussian-quadrature based fast maximum penalized likelihood (MPL) method for fitting CSH Cox models, where the unknown baseline hazards are approximated using M-splines. Our proposed method provides estimates of both the regression coefficients and approximated baseline hazards, where the non-negative constraint on the baseline hazards is respected. In this method, penalty functions are employed for two reasons: to regularize estimates of the baseline hazards and to ease the requirement for optimal location and number of knots^6^ needed for approximating the baseline hazards.

For competing risks data under interval censoring, there exist a few likelihood based methods, particularly Li^5^ and Joly et al.^7^ The latter is in fact for the illness-death model without covariates, but it can be implemented to competing risks. The method by Li^5^ allows covariates, but its optimization computations are achieved through an existing R nonlinear optimization function “nlminb,” which can be inefficient and slow. Another issue of Li^5^ is that it ignores some non-negative parameters of the approximated baseline hazards can be active, leading to possible unpleasant consequences such as negative variances. Our proposed method will address properly the issue of active constraints.

The remaining of this article is organized as follows. Section 2 defines the likelihood under competing risks CSH Cox models and with partly interval censoring. Section 3 provides the details on the computation of the MPL estimates of regression coefficients and baseline hazards and it also discusses how to automatically select the optimal smoothing parameter. Asymptotic results of the MPL estimates are presented in Section 4. Section 5 contains simulation results and also an application of our method to the ASPREE study. Finally, the concluding remarks are given in Section 6.

## 2. Model and likelihood

Partly interval censoring (e.g. Kim^4^) refers to the situation where observed survival times are flexible, and they can include event times, and also left, right or interval censoring times. In this article, we study competing risks CSH models with partly interval censoring. Particularly, we propose a Gaussian quadrature based penalized likelihood approach to estimate semiparametric CSH models, where both regression coefficients and baseline hazards are estimated.

Let $Y_{i}$ denote the event time for individual i , but due to censoring, $Y_{i}$ may not be fully observed. Partly interval censoring means that $Y_{i}$ can be fully observed or left-, right-, and interval-censored. For competing risks, when an event has occurred (i.e. an event time or left or interval censoring time is observed), the event is then linked with only one of the risks. When an event does not occur (i.e. a right censoring time is observed), there is no link with any of the risks. Therefore, we can define an indicator variable $\kappa_{ir}$ to link an event with risk r : $\kappa_{ir}=1$ if $Y_{i}$ is associated with risk r and $\kappa_{ir}=0$ for otherwise. Let the vector $\kappa_{i}=(\kappa_{i1},\ldots ,\kappa_{ig}{)}^{⊤}$ . When $Y_{i}$ is right-censored, then $\kappa_{i}=0_{g\times 1}$ , a zero vector with length g .

To accommodate interval censoring, we define $t_{i}^{L}$ and $t_{i}^{R}$ (where $t_{i}^{L}\leq t_{i}^{R}$ ) as a pair of times for each i such that $Y_{i}\in [t_{i}^{L},t_{i}^{R}]$ . If $t_{i}^{L}=t_{i}^{R}=t_{i}$ , then $Y_{i}$ is fully observed with $Y_{i}=t_{i}$ . If $t_{i}^{L}=0$ and $t_{i}^{R}$ is finite, then $Y_{i}$ is left censored at $t_{i}^{R}$ . If $t_{i}^{R}=+\infty$ and $t_{i}^{L}\neq 0$ , then $Y_{i}$ is right censored at $t_{i}^{L}$ . For other cases, $Y_{i}$ is interval censored. We also need indicators for censoring types. Let $\delta_{i},\delta_{i}^{R},\delta_{i}^{L}$ , and $\delta_{i}^{I}$ denote event, right, left, and interval censoring, respectively. Clearly, $\delta_{i}+\delta_{i}^{R}+\delta_{i}^{L}+\delta_{i}^{I}=1$ . We need both $\delta_{i},\delta_{i}^{R},\delta_{i}^{L},\delta_{i}^{I}$ , and $\kappa_{i}$ to define the event, censoring, and the associated risk when the event occurs. Let $\kappa_{i0}=1-\sum_{r=1}^{g}\kappa_{ir}$ . Clearly, a right-censored event can be represented by either $\kappa_{i0}=1$ or $\delta_{i}^{R}=1$ .

For each individual i , the observed values can be denoted as $\left(t_{i}^{L},t_{i}^{R},\delta_{i},\delta_{i}^{R},\delta_{i}^{L},\delta_{i}^{I},\kappa_{i}^{⊤},X_{i}^{⊤}\right)$ , where i=1,…,n ( n denotes the sample size), and $X_{i}$ is a vector for values of p covariates. Let $h_{r}(t)$ denote the CSH for risk r , which quantifies the chance of an event with risk r occurring at time t when an event of any risk has not yet occurred up to time t . In this article, we consider semi-parametric CSH Cox regression models. Thus, for each risk r , r=1,…,g , we consider the following CSH Cox model:

(1) $$h_{r}(t|X_{i})=h_{0r}(t)\exp\;\{X_{i}^{⊤}\beta_{r}\}$$

where $\beta_{r}=(\beta_{r1},\ldots ,\beta_{rp}{)}^{⊤}$ is a p -vector of regression coefficients of risk r and $h_{0r}(t)\geq 0$ is an unspecified baseline hazard function for risk r .

Let $H_{r}(t)=\int_{0}^{t}h_{r}(w)dw$ , and this function represents the cumulative cause-specific hazard at time t . From all $h_{r}(t)$ , the overall hazard for an event is given by $h(t)=\sum_{r=1}^{g}h_{r}(t),$ and the overall survival can be computed from $S(t)=\prod_{r=1}^{g}S_{r}(t),$ where $S_{r}(t)=\exp\;\{-H_{r}(t)\}$ .

In competing risks analysis, a cumulative incidence function (CIF), which quantifies the probability of an event with a particular risk occurring before a time t , is often computed and reported. Let ${\bar{F}}_{r}(t)$ denote the CIF for competing risk r at time t , and this function is given by

(2) $${\bar{F}}_{r}(t)=\int_{0}^{t}h_{r}(w)S(w)dw$$

The cumulative distribution function for the event at time t is $F(t)=\sum_{r=1}^{g}{\bar{F}}_{r}(t)$ , implying that S(t) can also be recovered from ${\bar{F}}_{r}(t)$ through $S(t)=1-\sum_{r=1}^{g}{\bar{F}}_{r}(t)$ .

Since each $h_{0r}(t)$ is non-parametric, it is an infinite-dimensional parameter. Estimation of $h_{0r}(t)$ from a finite number of observations is an ill-posed problem. One common approach to alleviate this issue is to approximate $h_{0r}(t)$ using a finite number of non-negative basis functions, where the number of basis functions is allowed to vary with the sample size at a slow rate. This method is known as the method of sieves.^8^ Using basis functions, the approximated $h_{0r}(t)$ is given by

(3) $$h_{0r}(t)=\sum_{u=1}^{m_{r}}\theta_{ru}\psi_{ru}(t)$$

where $\psi_{ru}(t)\geq 0$ are basis functions (e.g. Ma et al.^9^ and Joly et al.^10^). Note both $m_{r}$ and $\psi_{ru}(t)$ are associated with risk r . Some examples of basis functions include M-splines (e.g. Ramsay^11^), Gaussian densities (e.g. Ma et al^9^ and Li and Ma^12^) or even indicator functions. The conventional non-parametric maximum likelihood estimate (NPMLE) of a baseline hazard (e.g. Anderson et al.^13^) is equivalent to adopting indicator basis functions using event times. We can now enforce the constraint $h_{0r}(t)\geq 0$ more easily by $\theta_{ru}\geq 0$ for all u . We comment that although a polynomial function can be used to approximate $h_{0r}(t)$ but is not ideal when compared with splines. To capture complex nonlinearities in a baseline hazard, a polynomial may require a high order, making it less flexible. In contrast, splines are more efficient as they utilize low-order polynomials between knots, allowing them to capture local nonlinear patterns more effectively.

Let $\theta_{r}=(\theta_{r1},\ldots ,\theta_{rm_{r}}{)}^{⊤}$ . For each i , we assume the censoring interval $\left(t_{i}^{L},t_{i}^{R}\right)$ is independent of the event time $Y_{i}$ given the covariates. Let vectors $\beta =(\beta_{1}^{⊤},\ldots ,\beta_{g}^{⊤}{)}^{⊤}$ and $\theta =(\theta_{1}^{⊤},\ldots ,\theta_{g}^{⊤}{)}^{⊤}$ . The length of β is gp and the length of θ is $\sum_{r=1}^{g}m_{r}$ . The log-likelihood from the partly interval censored competing risks data is

(4) $$\begin{array}{rl} l(\beta ,\theta ) & =\sum_{i=1}^{n}\sum_{r=1}^{g}\{\kappa_{ir}[\delta_{i}\log\;h_{r}(t_{i})+\delta_{i}^{L}\log\;({\bar{F}}_{r}(t_{i}^{R}))+\delta_{i}^{I}\log\;({\bar{F}}_{r}(t_{i}^{R})-{\bar{F}}_{r}(t_{i}^{L}))]\\ & \;-\left(\delta_{i}(1-\kappa_{i0})+\kappa_{i0}\right)H_{r}(t_{i})-\delta_{i}^{R}H_{r}(t_{i}^{L})\} \end{array}$$

When there are neither left nor interval censoring times (so only events and right censoring), expression (4) will contain only $h_{r}(t)$ and $H_{r}(t)$ and the log-likelihood is fully separated for different r . Thus, in this context, (4) can be maximized separately for each risk r , similar to the partial likelihood method for competing risks, by setting the event times from other risks as the right censoring times. However, when left or interval censoring presents, the log-likelihood in (4) is in general difficult to maximize. This is because the left or interval censoring will cause the log-likelihood to include the CIFs ${\bar{F}}_{r}(t)$ , while each CIF demands cause-specific survival functions from all the risks, which means parameters of one competing risk depend on the parameters of all the other risks. In the next section, we will discuss how to estimate β and θ by maximizing a penalized log-likelihood function where penalty functions are used to restrain the θ estimate.

## 3. Penalized likelihood estimation

### 3.1. Computation

We wish to estimate parameters β and θ by the maximum penalized likelihood (MPL) approach where penalty functions are used for two reasons: (i) to smooth the estimates of the baseline hazards $h_{0r}(t)$ ; and (ii) to make the $h_{0r}(t)$ estimates less sensitive to the number of location of knots. Specifically, the MPL estimates of β and θ are given by

(5) $$(\hat{\beta },\hat{\theta })=\underset{\beta ,\theta }{\mathrm{argmax}}\;\left\{\Phi (\beta ,\theta )=l(\beta ,\theta )-\sum_{r=1}^{g}\lambda_{r}J_{r}(\theta_{r})\right\}$$

subject to θ≥0 . Here, $\lambda_{r}>0$ are smoothing parameters and $J_{r}(\cdot )$ are penalty functions used to restrain $\theta_{r}$ . A common choice of the penalty $J_{r}(\cdot )$ is the roughness penalty,^14^ given by

(6) $$J_{r}(\theta_{r})=\int (h_{0r}^{\prime \prime }(t){)}^{2}dt=\theta_{r}^{⊤}R_{r}\theta_{r}$$

where matrix $R_{r}$ has the dimension of $m_{r}\times m_{r}$ and its (u,v) -th element is $\int \psi_{ru}^{\prime \prime }(t)\psi_{rv}^{\prime \prime }(t)dt$ .

This is a difficult constrained optimization problem. Employing an R or MATLAB optimization solver to find the solutions is not ideal. This is because (i) these solvers can be limited on the number of constraints (thus the size of θ ) they can handle; (ii) a solver can be slower, meaning an unnecessary long computation time. Li,^5^ for example, reported that when sample size n=500 it took close to 10  min for the proposed R program to find the constrained optimal solution under a fixed smoothing value, and this computational time extended to 7  h when leave-one-out cross validation was used to select the smoothing parameter. In this article, we aim to develop a practically feasible computation procedure to estimate β , θ and also to estimate the smoothing values $\lambda_{1},\ldots ,\lambda_{g}$ . We first discuss how to estimate β and θ (where θ≥0 ) when the smoothing parameters are fixed. Then, in Section 3.2, we will develop a marginal likelihood based approach to estimate the smoothing parameters.

For given smoothing parameters $\lambda_{1},\ldots ,\lambda_{g}$ , the Karush-Kuhn-Tucker (KKT) necessary conditions for the constrained optimization problem (5) are defined by the following set of equations: for r=1,…,g , $u=1,\ldots ,m_{r}$ , and j=1,…,p ,

$$\begin{array}{rl} & \frac{\partial \Phi (\beta ,\theta )}{\partial \beta_{rj}}=0\\ & \frac{\partial \Phi (\beta ,\theta )}{\partial \theta_{ru}}=0,\;\text{if}\;\theta_{ru}>0\\ & \frac{\partial \Phi (\beta ,\theta )}{\partial \theta_{ru}}<0,\;\text{if}\;\theta_{ru}=0 \end{array}$$

We solve these KKT equations using an alternating algorithm, in which β and θ are updated alternately. Specifically, we use a quasi-Newton step with line search to update β in each iteration, and then adopt a multiplicative-iterative (MI) step (e.g. Chan and Ma^15^) with line search to update θ , ensuring that the updated θ values are non-negative.

The derivative of Φ with respect to β or θ (details of these derivatives are given in Supplemental Material of this article) involves the derivative of ${\bar{F}}_{q}(t_{i})$ , q=1,…,g , with respect to β or θ , where the latter derivatives are given by

(7) $$\begin{array}{rl} \frac{\partial {\bar{F}}_{q}(t_{i})}{\partial \beta_{rj}} & =A_{qri}(t_{i})x_{ij} \end{array}$$

(8) $$\begin{array}{rl} \frac{\partial {\bar{F}}_{q}(t_{i})}{\partial \theta_{ru}} & =(B_{qrui1}(t_{i})-B_{qrui2}(t_{i}))e^{X_{i}\beta_{r}} \end{array}$$

Within the expressions in (7) and (8), we have

$$\begin{array}{rl} & A_{qri}(t)=\int_{0}^{t}h_{q}(w|X_{i})S(w|X_{i})(1_{\left\{q=r\right\}}-H_{r}(w|X_{i}))dw\\ & B_{qrui1}(t)=\int_{0}^{t}1_{\left\{q=r\right\}}\psi_{ru}(w)S(w|X_{i})dw\\ & B_{qrui2}(t)=\int_{0}^{t}h_{q}(w|X_{i})\Psi_{ru}(w)S(w|X_{i})dw \end{array}$$

where $\Psi_{ru}(t)=\int_{0}^{t}\psi_{ru}(w)dw$ , representing the cumulative basis function. Note that the amount of these quantities can be large, depending on n , g , and m . More specifically, the size of $A_{qri}(t_{i})$ , $B_{qrui1}(t_{i})$ , and $B_{qrui2}(t_{i})$ are, respectively, g×g×n , g×m×n , and g×g×m×n . Unless there are closed-form expressions for the integrals, evaluation of these quantities is a tremendous computational burden, let alone that these quantities need to be re-evaluated each time when β or θ are updated. Therefore, to design a fast algorithm, these integrals must be approximated so that fast evaluations become possible. Towards this, we adopt the Gaussian quadrature approximations; see, for example, Golub and Welsch.^16^

Consider a general integral $c=\int_{0}^{a}e(w)dw.$ Let $\kappa_{1},\ldots ,\kappa_{V}$ be predetermined quadrature points and $w_{1},\ldots ,w_{V}$ be the corresponding weights, then the Gaussian quadrature approximation to this integral is given by

(9) $$c=\sum_{v=1}^{V}e(\kappa_{v})w_{v}$$

Computation of (9) is typically straightforward and yields accurate results. The quadrature points and weights are determined by the chosen approach, such as the Legendre-Gauss quadrature employed in this article.

Before explaining the algorithm, we first introduce some notations. In this article, we let $a^{\left(k\right)}$ represent a parameter estimate at iteration k . Let $\left[a{]}^{+}=\max\{0,a\right\}$ and $\left[a{]}^{-}=\min\{0,a\right\}$ . We employ an alternating algorithm where each iteration comprises a pseudo Newton step to update β and a MI step to update θ . Thus, we call this the Newton–MI algorithm. Convergence properties of this algorithm can be found by Chan and Ma.^15^ In iteration k+1 , $\beta_{r}$ and $\theta_{r}$ , r=1,…,g , are updated by pseudo Newton and MI schemes, respectively:

(10) $$\begin{array}{rl} \beta_{r}^{\left(k+1\right)} & =\beta_{r}^{\left(k\right)}+\omega_{1r}^{\left(k\right)}[X^{T}V_{r}({\tilde{\beta }}_{\left[r\right]}^{\left(k\right)},\theta^{\left(k\right)})X{]}^{-1}\frac{\partial \Phi ({\tilde{\beta }}_{\left[r\right]}^{\left(k\right)},\theta^{\left(k\right)})}{\partial \beta_{r}} \end{array}$$

(11) $$\begin{array}{rl} \theta_{r}^{\left(k+1\right)} & =\theta_{r}^{\left(k\right)}+\omega_{2r}^{\left(k\right)}S_{r}(\beta^{\left(k+1\right)},{\tilde{\theta }}_{\left[r\right]}^{\left(k\right)})\frac{\partial \Phi (\beta^{\left(k+1\right)},{\tilde{\theta }}_{\left[r\right]}^{\left(k\right)})}{\partial \theta_{r}} \end{array}$$

where $\omega_{1r}$ and $\omega_{2r}$ are the line search step sizes. In (10), $V_{r}(\beta ,\theta )$ is a diagonal matrix given by

$$\begin{array}{rl} V_{r}(\beta ,\theta ) & =\text{diag}(\left(\delta_{i}(1-\kappa_{i0})+\kappa_{i0}\right){\tilde{H}}_{r}(t_{i})+\delta^{L}\sum_{q}\kappa_{iq}\frac{A_{qri}^{2}(t_{i})}{{\bar{F}}_{q}^{2}(t_{i})}\\ & \;+\delta_{i}^{I}\sum_{q}\kappa_{iq}\frac{(A_{qri}(t_{i}^{R})-A_{qri}(t_{i}^{L}){)}^{2}}{({\bar{F}}_{q}(t_{i}^{R})-{\bar{F}}_{q}(t_{i}^{L}){)}^{2}}) \end{array}$$

and ${\tilde{\beta }}_{\left[r\right]}^{\left(k\right)}=(\beta_{<r}^{(k+1)\;⊤},\beta_{\geq r}^{(k)\;⊤}{)}^{⊤}$ , where $\beta_{<r}=(\beta_{1}^{⊤},\ldots ,\beta_{r-1}^{⊤}{)}^{⊤}$ and $\beta_{\geq r}=(\beta_{r}^{⊤},\ldots ,\beta_{g}^{⊤}{)}^{⊤}$ . In (11), matrix $S_{r}(\beta ,\theta )$ is also a diagonal matrix, given by: $S_{r}(\beta ,\theta )=\text{diag}(\theta_{ru}/(\tau_{ru}+\varepsilon ))$ , where

$$\begin{array}{rl} \tau_{ru} & =\sum_{i}e^{X_{i}\beta_{r}}(\left(\delta_{i}(1-\kappa_{i0})+\kappa_{i0}\right)\Psi_{ru}(t_{i})+\delta^{L}\sum_{q}\kappa_{iq}\frac{B_{qrui2}(t_{i})}{{\bar{F}}_{q}(t_{i})}\\ & \;+\delta^{I}\sum_{q}\kappa_{iq}\frac{B_{qrui1}(t_{i}^{L})+B_{qrui2}(t_{i}^{R})}{{\bar{F}}_{q}(t_{i}^{R})-{\bar{F}}_{q}(t_{i}^{L})})+[R_{r}\theta_{r}{]}^{+} \end{array}$$

and ${\tilde{\theta }}_{\left[r\right]}^{\left(k\right)}$ is defined similar to ${\tilde{\beta }}_{\left[r\right]}^{\left(k\right)}$ . It is not difficult to verify that when adopting the MI scheme to update θ , for any r , if $\theta_{r}^{\left(k\right)}\geq 0$ then we have $\theta_{r}^{\left(k+1\right)}\geq 0$ . From the above expressions, we can see that fast evaluation of the $A_{qri}$ , $B_{qrui1}$ , $B_{qrui2}$ , and ${\bar{F}}_{q}$ function values is a key requirement for our algorithm (or any computing procedure to solve this problem) to be practically useful.

We comment that line search step sizes $\omega_{1}^{\left(k\right)}$ and $\omega_{2}^{\left(k\right)}$ can be fast computed using, for example, the Armijo’s inexact line search method (e.g. Luengerger^17^). Following the arguments of Chan and Ma,^15^ it can be demonstrated that, under certain regularity conditions, this Newton–MI algorithm produces a convergent sequence $\left\{(\beta^{\left(k\right)},\theta^{\left(k\right)})\right\}$ , and moreover, the solution obtained at the convergence satisfies the KKT conditions.

### 3.2. Automatic smoothing parameter selection

Our penalized likelihood approach includes an essential feature: automatic selection of the smoothing parameter. This procedure is especially valuable for users who may not be familiar with the penalized likelihood method. This smoothing parameter selection method is devised by treating the penalty functions as log-prior density functions and views $\lambda_{r}$ as related to the variances of these prior distributions, and these variances can then be estimated by maximization of a marginal likelihood.^18,19^ The roughness penalty on $h_{0r}(t)$ can be expressed as a quadratic function of $\theta_{r}$ ; see (6). Thus, we can relate the penalty $\lambda_{r}J_{r}(\theta_{r})$ to the log density of $N(0_{m\times 1},\sigma_{r}^{2}R_{r}^{-1})$ , where $\sigma_{r}^{2}=1/(2\lambda_{r})$ . The corresponding log-posterior density is

(12) $$l_{p}(\beta ,\theta )=-\frac{m}{2}\sum_{r=1}^{g}\log\;\sigma_{r}^{2}+l(\beta ,\theta )-\sum_{r=1}^{g}\frac{1}{2\sigma_{r}^{2}}\theta_{r}^{T}R_{r}\theta_{r}$$

The log-marginal likelihood for $\sigma_{1}^{2},\ldots ,\sigma_{g}^{2}$ (after integrating out β and θ ) is

(13) $$l_{m}(\sigma_{1}^{2},\ldots ,\sigma_{g}^{2})=-\frac{m}{2}\sum_{r=1}^{g}\log\;\sigma_{r}^{2}+\log\;\int \exp\;(l(\beta ,\theta )-\sum_{r=1}^{g}\frac{1}{2\sigma_{r}^{2}}\theta_{r}^{T}R_{r}\theta_{r})d\beta d\theta$$

To overcome the infeasibility of directly maximizing the high-dimensional integral in equation (13), we employ Laplace’s method as an approximation technique. Let $\hat{\beta }$ and $\hat{\theta }$ denote, respectively, the β and θ values maximizing $l_{p}(\beta ,\theta )$ with fixed $\sigma_{r}^{2}$ values (so that equivalent to Φ(β,θ) with fixed smoothing values), then using Laplace’s approximation we have

(14) $$l_{m}(\sigma_{1}^{2},\ldots ,\sigma_{g}^{2})\approx -\frac{m}{2}\sum_{r=1}^{g}\log\;\sigma_{r}^{2}+l(\hat{\beta },\hat{\theta })-\frac{1}{2}\sum_{r=1}^{g}\left[{\hat{\theta }}^{T}R_{r}{\hat{\theta }}_{r}/\sigma_{r}^{2}-\log\;\left|{\hat{G}}_{r}+Q(\sigma_{r}^{2})\right|\right]$$

where ${\hat{G}}_{r}=-\partial^{2}l(\hat{\beta },{\hat{\theta }}_{r})/\partial \beta \partial \theta_{r}$ and matrix

$$Q(\sigma_{r}^{2})=\left(\begin{array}{cc} 0_{p\times p} & 0_{p\times m_{r}}\\ 0_{m_{r}\times p} & R_{r}/\sigma_{r}^{2} \end{array}\right)$$

Maximizing (14) gives the solutions satisfying

(15) $${\hat{\sigma }}_{r}^{2}=\frac{{\hat{\theta }}_{r}^{T}R_{r}{\hat{\theta }}_{r}}{m_{r}-{\hat{\nu }}_{r}}$$

where ${\hat{\nu }}_{r}$ is given by

$${\hat{\nu }}_{r}=\text{tr}\left\{{\left({\hat{G}}_{r}+Q({\hat{\sigma }}_{r}^{2})\right)}^{-1}Q({\hat{\sigma }}_{r}^{2})\right\}$$

If active constraints of $\theta_{r}\geq 0$ are taken into consideration, then ${\hat{\nu }}_{r}$ can be modified using the technique specified in the next section, where a matrix $U_{r}$ is constructed, similar to (17) and with the dimension of $\left(m_{r}+p)\times (m_{r}+p-d_{r}\right)$ . Here, $d_{r}$ is the number of active constraints of $\theta_{r}\geq 0$ . The modified $\nu_{r}$ is given by

$${\hat{\nu }}_{r}=\text{tr}\left\{U_{r}{\left(U_{r}^{T}({\hat{G}}_{r}+Q({\hat{\sigma }}_{r}^{2}))U_{r}\right)}^{-1}U_{r}^{T}Q({\hat{\sigma }}_{r}^{2})\right\}$$

We note that the estimate of $\sigma_{r}^{2}$ provided in equation (15) is only an approximate solution for maximizing the approximate marginal likelihood (14). These approximations will make it difficult to obtain asymptotic properties for ${\hat{\sigma }}_{r}^{2}$ . However, this lack of $\sigma_{r}^{2}$ estimate properties does not pose an issue as our primary focus lies on the estimation of β and $h_{r0}(t)$ , for which consistent results are provided in Theorem 1.

Note that both β and θ depend on all $\sigma_{r}^{2}$ , which means that their estimation requires different iterative procedures. Specifically, our algorithm involves inner and outer iterations. In each round of inner–outer iterations, β and θ are first updated in inner iterations using the Newton–MI algorithm described above with $\sigma_{r}^{2}$ (or equivalently $\lambda_{r}$ ) fixed at its current value. Then, with β and θ fixed at their current estimates, all $\sigma_{r}^{2}$ are updated using (15) in the outer iterations. This process continues until the degrees-of-freedom $\nu_{r}$ are stabilized, which means that the differences between their values in consecutive iterations are less than 1. The simulation study reported in Section 5 reveals that this procedure converges quickly, where the M-splines basis functions are adopted.

Cross-validation (CV) is another commonly adopted approach for estimating the smoothing parameters, and it aims at minimizing prediction errors. As explained by Wood,^19^ for semiparametric generalized linear model estimation, marginal likelihood might be preferable to CV due to its resistance to overfitting, lower smoothing parameter variability, and reduced tendency towards multiple minima. Our experience reveals that the proposed approximate marginal likelihood approach has much less computational burden than the CV or the generalized CV method.

## 4. Large sample properties

In this section, we provide some large sample results for the MPL estimates of the CSH Cox model. Let $\eta =(\theta^{⊤},\beta^{⊤}{)}^{⊤}$ and let $\mu_{rn}=\lambda_{r}/n$ . The penalized log-likelihood in (5) can be expressed as

(16) $$\Phi (\eta )=\sum_{i=1}^{n}(l_{i}(\eta )-\sum_{r=1}^{g}\mu_{rn}J_{r}(\eta ))$$

where $l_{i}(\eta )$ can be obtained from (4) and $J_{r}(\eta )=J_{r}(\theta_{r})$ . When maximizing Φ(η) subject to θ≥0 , it is common for some estimated elements of θ to be zero, which means they are active constraints. Ignoring active constraints may result in negative asymptotic variances.

### 4.1. Asymptotics when all $m_{r}\to \infty$ and all $\mu_{rn}\to 0$

Consistency of the MPL estimates will be considered here under the assumption that, when n→∞ , all $m_{r}\to \infty$ (but $m_{r}/n\to 0$ ) and all $\mu_{rn}\to 0$ . We assume specifically that $\mu_{rn}=o(n^{-1/2})$ . For a given sample with size n , let $\hat{\theta }$ be the MPL estimate of θ from this sample and the baseline hazards for cause r is ${\hat{h}}_{0r}(t)=\sum_{i=1}^{m}{\hat{\theta }}_{ru}\psi_{ru}(t)$ .

Consistency of $\hat{\beta }$ and ${\hat{h}}_{0r}(t)$ ( r=1,…,g ) can be established using similar arguments as in the Supplemental Material of Ma et al.^9^

Theorem 1 Assume Assumptions A1–A3 stated in the Supplemental Material hold and assume all $h_{r0}(t)$ have the first c≥1 derivatives. Let $a=\min_{i}\{t_{i}\}$ and $b=\max_{i}\{t_{i}\}$ . Take $\mu_{rn}=o(n^{-1/2})$ and $m_{r}=n^{\rho_{r}}$ , where $1/(2(1+c))\leq \rho_{r}\leq 1/2c$ , then when n→∞ :

1.
$\|\hat{\beta }-\beta_{0}\|\to 0$ .
2. For all r , $\underset{t\in [a,b]}{\sup}|{\hat{h}}_{0r}(t)-h_{0r}^{0}(t)|\overset{a.s.}{\to }0$ .

Here, $\beta_{0}$ and $h_{0r}^{0}(t)$ denote the true regression coefficient vector and true baseline hazard for risk r , respectively.

The proofs of these results can be given directly by following the proofs that appear in the Supplemental Material of Ma et al.^9^

The results in Theorem 1 establish consistency for both $\hat{\beta }$ and ${\hat{h}}_{0r}(t)$ , but they have limited practical usefulness because (i) they do not specify the asymptotic covariance of ${\hat{h}}_{0r}(t)$ , and (ii) in data analysis, n is always finite and therefore neither $m_{r}$ ’s are infinity nor $\mu_{rn}$ ’s are zero.

In the next section, we develop a large sample normality property for $\hat{\beta }$ and $\hat{\theta }$ , but with fixed $m_{r}$ ’s and nonzero $\mu_{rn}$ ’s. This result also addresses the problem of active constraints.

### 4.2. Large sample normality when all $m_{r}$ are finite

In practice, since the $m_{r}$ ’s are never infinite, it is necessary to develop large sample normality when $m_{r}$ ’s are finite. Let $\beta_{0}$ and $\theta_{0}$ be the true parameters corresponding to the fixed $m_{r}$ ’s and $\lambda_{r}$ ’s. For each constraint $\theta_{r}\geq 0$ , it is possible that some components of the MPL estimate of $\theta_{r}$ are actively constrained. Our large sample normality result will be able to accommodate active constraints. Asymptotic properties for constrained maximum likelihood estimates under parametric models are available in, for example, Moore et al.^20^ We will follow this reference in the following discussions.

Assume, without loss of generality, that the first $d_{r}$ elements of each $\theta_{r}\geq 0$ constraint are active in the MPL solution. Define matrix $U_{r}$ as

(17) $$\begin{array}{r} U_{r}=[0_{(m_{r}+p-d_{r})\times d_{r}},I_{\left(m_{r}+p-d_{r})\times (m_{r}+p-d_{r}\right)}{]}^{⊤} \end{array}$$

where 0 is a matrix of zeros and I is an identity matrix. Note that $U_{r}^{⊤}U_{r}=I_{\left(m_{r}+p-d_{r})\times (m_{r}+p-d_{r}\right)}$ . Let a block-diagonal matrix $U=\text{diag}(U_{1},\ldots ,U_{g})$ , then $U^{⊤}U$ is also an identity matrix with $\sum_{r=1}^{g}m_{r}+gp-\sum_{r=1}^{n}d_{r}$ diagonals. We need the following assumptions before giving the large sample normality result.

Theorem 2 Assume Assumptions A1, A3–A7 stated in the Supplemental Material hold. Assume in each $\theta_{r}\geq 0$ there are $d_{r}$ active constraints, r=1,…,g . Corresponding to these active constraints, the matrix $U_{r}$ is defined similar to (17) and a block-diagonal matrix U matrix is constructed as above. Then, when n is large, $\sqrt{n}(\hat{\eta }-\eta_{0})$ is approximately multivariate normal with 0 mean vector and covariance matrix $\tilde{F}(\eta_{0}{)}^{-1}G(\eta_{0})[\tilde{F}(\eta_{0}{)}^{-1}{]}^{⊤}$ , where $\tilde{F}(\eta {)}^{-1}=U(U^{⊤}F(\eta )U{)}^{-1}U^{⊤}$ .

Again, the proofs of these results can be obtained by directly following the proofs of the similar results that appear in the Supplemental Material of Ma et al.^9^

We remark that matrix $\tilde{F}(\eta {)}^{-1}$ is easy to compute. This is because $U^{⊤}F(\eta )U$ is simply given by deleting the rows and columns of F(η) where these row and column indices are determined by the positions of the active constraints. Then $\tilde{F}(\eta {)}^{-1}$ is obtained by adding zeros to the inverse of $U^{⊤}F(\eta )U$ at the positions of the deleted rows and columns. In applications, the unknown $\eta_{0}$ can be replaced by the MPL estimates $\hat{\eta }$ . The simulation results reported in Section 5 demonstrate that standard errors of the MPL estimates are generally accurate.

## 5. Results

### 5.1. Simulation

Monte Carlo simulations were undertaken to compare the performance of the MPL method with an ad-hoc method commonly used in practice denoted as midpoint Cox. This involves applying a Cox regression to each risk separately, while right censoring all other risks. For interval censored observations, the midpoint of the interval was treated as an exact observed time, and for left censored data, the observed time was divided by 2. We generated survival times, T , from the following Weibull distribution for two risks ( r=1,2 ).

(18) $$\begin{array}{r} h_{0r}\left(t\right)=\lambda_{r}\rho_{r}t^{\rho_{r}-1} \end{array}$$

Three studies were conducted. In study 1, T was generated from the Weibull distribution with scale parameters $\lambda_{1}=1$ and $\lambda_{2}=0.5$ , and the shape parameters $\rho_{1}=\rho_{2}=3.$ Covariates from two variables were simulated from $x_{i1}∼N(0,1)$ and $x_{i2}∼N(0,1)$ , with coefficient values $\beta_{11}=-1,\beta_{12}=0.5$ for risk 1 , and $\beta_{21}=1,\beta_{22}=-0.5$ for risk 2. In study 2, T was generated from the same scale and shape parameters, as well as covariates from study 1. The coefficient values were $\beta_{11}=1,\beta_{12}=0.5$ for risk 1 , and $\beta_{21}=0.5,\beta_{22}=0.5$ for risk 2. In study 3, T was generated from $\lambda_{1}=1$ and $\lambda_{2}=0.5$ with shape parameter $\rho_{1}=\rho_{2}=2.$ The covariates from two variables were simulated from $x_{i1}∼N(0,1)$ and a binary variable with $x_{i2}∼B(1,0.5).$ The coefficient values were $\beta_{11}=-1,\beta_{12}=0.5$ for risk 1 , and $\beta_{21}=1,\beta_{22}=-0.5$ for risk 2.

Observed survival times $\left(t_{i}^{L},t_{i}^{R}\right)$ for right censored, event, left censored, and interval censored times were generated as follows. Let $T_{i}$ denote a simulated event time as described, $\pi^{E}$ represent a chosen proportion for event times, $U_{i}^{L}$ and $U_{i}^{E}$ are generated from unif(0,1) and $U_{i}^{R}$ from $\text{unif}(U_{i}^{L},1)$ . Let $\gamma_{L}$ and $\gamma_{R}$ (with $\gamma_{L}\leq \gamma_{R}$ ) be two positive scalars. The width of randomly generated intervals can be increased by selecting γ values that are further apart. If $U_{i}^{E}<\pi^{E}$ then we have an event time so that $t_{i}^{L}=t_{i}^{R}=T_{i}$ ; otherwise, if $\gamma_{L}U_{i}^{L}\leq T_{i}\leq \gamma_{R}U_{i}^{R}$ we have interval censoring with $t_{i}^{L}=\gamma_{L}U_{i}^{L}$ and $t_{i}^{R}=\gamma_{R}U_{i}^{R}$ ; if $T_{i}<\gamma_{L}U_{i}^{L}$ we have left censoring with $t_{i}^{L}=0$ and $t_{i}^{R}=\gamma_{L}U_{i}^{L}$ and if $\gamma_{R}U_{i}^{R}<T_{i}$ we have right censoring with $t_{i}^{L}=\gamma_{R}U_{i}^{R}$ and $t_{i}^{R}=\infty$ . The probability of risk group membership, $p_{ir}$ , is given by a ratio of the hazards by:

(19) $$p_{ir}=\frac{h_{r}\left(t\right)}{h\left(t\right)}$$

We generated a random variable $W_{i}$ from unif(0,1) , and if $W_{i}<p_{i1}$ then i belongs to risk group 1, else i belongs to risk group 2.

The simulation study was designed to examine the estimation method under varying sample sizes and censoring proportions. We ran Monte Carlo simulations with sample sizes of n = 200 and 1000. For all studies, the event proportion was fixed at $\pi^{E}=5\%$ . Within each study, we examined two sets of censoring proportions for each sample size. The first set was an approximate right censoring proportion of 47.5% , and an approximate interval censoring (including left censoring) of 47.5% . These correspond to gamma values of $\gamma_{L}=0.5$ and $\gamma_{R}=0.91$ for study 1, $\gamma_{L}=0.5$ and $\gamma_{R}=1.47$ for study 2, and $\gamma_{L}=0.5$ and $\gamma_{R}=0.74$ for study 3. The second set of censoring proportions was an approximate right censoring proportion of 20% , and an approximate interval censoring (including left censoring) of 75% . These correspond to gamma values of $\gamma_{L}=0.5$ and $\gamma_{R}=1.34$ for study 1, $\gamma_{L}=0.5$ and $\gamma_{R}=1.64$ for study 2, and $\gamma_{L}=0.5$ and $\gamma_{R}=1.27$ for study 3. Bias was calculated by taking the average of differences between the parameter estimates and the true values. Asymptotic standard deviations (std asymp) was calculated by taking the average of the estimated standard deviations. Monte Carlo standard deviations (std mc) was calculated by taking the standard deviation of the Monte Carlo parameter estimates. Coverage probability (cp) was calculated by taking the proportion of Monte Carlo runs where the true parameter was contained within the confidence interval.

**Table 1.** Cox (midpoint *t*) and MPL regression parameter estimation for study 1.

				$\beta_{11}=-1$		$\beta_{12}=0.5$		$\beta_{21}=1$		$\beta_{22}=-0.5$	
*n*	int cens (%)	right cens (%)		Cox	MPL	Cox	MPL	Cox	MPL	Cox	MPL
200	47.5	47.5	bias	−0.053	0.033	0.033	−0.01	0.019	−0.048	−0.002	0.032
			std asymp	0.148	0.169	0.134	0.146	0.191	0.208	0.173	0.182
			std mc	0.154	0.17	0.142	0.15	0.204	0.22	0.181	0.189
			cov prob	0.92	0.957	0.927	0.949	0.937	0.945	0.936	0.95
200	75	20	bias	−0.223	0.024	0.114	−0.012	0.121	−0.038	−0.054	0.026
			std asymp	0.119	0.173	0.109	0.145	0.15	0.203	0.136	0.17
			std mc	0.135	0.176	0.115	0.144	0.165	0.209	0.142	0.175
			cov prob	0.502	0.947	0.792	0.954	0.828	0.952	0.912	0.939
1000	47.5	47.5	bias	−0.081	0.016	0.039	−0.01	0.044	−0.013	−0.02	0.009
			std asymp	0.064	0.074	0.058	0.064	0.081	0.09	0.074	0.078
			std mc	0.066	0.076	0.06	0.066	0.081	0.089	0.074	0.08
			cov prob	0.721	0.941	0.873	0.945	0.912	0.948	0.946	0.942
1000	75	20	bias	−0.245	0.02	0.119	−0.012	0.141	−0.015	−0.07	0.01
			std asymp	0.056	0.078	0.047	0.064	0.064	0.091	0.059	0.075
			std mc	0.056	0.078	0.049	0.066	0.07	0.092	0.058	0.074
			cov prob	0.01	0.95	0.299	0.946	0.4	0.945	0.763	0.955

*Note:* MPL: maximum penalized likelihood. Cox (midpoint *t*) refers to the midpoint of interval censored times being treated as event times in a Cox regression; std asymp: asymptotic standard deviations; std mc: Monte Carlo standard deviations; cov prob: coverage probability.

**Table 2.** Cox (midpoint *t*) and MPL regression parameter estimation for study 2.

				$\beta_{11}=1$		$\beta_{12}=0.5$		$\beta_{21}=0.5$		$\beta_{22}=0.5$	
*n*	int cens (%)	right cens (%)		Cox	MPL	Cox	MPL	Cox	MPL	Cox	MPL
200	47.5	47.5	bias	0.12	−0.002	0.072	0.003	0.094	0.039	0.05	0.008
			std asymp	0.134	0.158	0.125	0.14	0.201	0.211	0.191	0.198
			std mc	0.141	0.158	0.129	0.142	0.2	0.219	0.202	0.211
			cov prob	0.822	0.953	0.891	0.951	0.93	0.931	0.931	0.938
200	75	20	bias	0.39	0.02	0.228	0.011	0.341	0.069	0.199	0.028
			std asymp	0.103	0.158	0.1	0.141	0.148	0.181	0.147	0.171
			std mc	0.111	0.164	0.106	0.143	0.148	0.186	0.147	0.174
			cov prob	0.073	0.931	0.371	0.949	0.361	0.923	0.696	0.939
1000	47.5	47.5	bias	0.136	−0.022	0.076	−0.015	0.101	0.016	0.06	0.007
			std asymp	0.058	0.071	0.055	0.063	0.087	0.095	0.082	0.087
			std mc	0.061	0.074	0.057	0.065	0.086	0.097	0.081	0.086
			cov prob	0.346	0.936	0.704	0.941	0.784	0.949	0.879	0.956
1000	75	20	bias	0.398	−0.009	0.23	−0.007	0.342	−0.023	0.203	−0.017
			std asymp	0.045	0.079	0.044	0.067	0.065	0.095	0.063	0.081
			std mc	0.048	0.085	0.046	0.068	0.063	0.095	0.063	0.082
			cov prob	0	0.932	0.001	0.939	0.001	0.951	0.111	0.941

*Note:* MPL: maximum penalized likelihood. Cox (Midpoint *t*) refers to the midpoint of interval censored times being treated as event times in a Cox regression; std asymp: asymptotic standard deviations; std mc: Monte Carlo standard deviations; cov prob: coverage probability.

In studies 1 and 2, bias was < 0.1 and lower for the majority of MPL results compared with the Cox regression with midpoint *t* (Tables 1 and 2). The asymptotic standard errors for the MPL were close to its Monte Carlo estimates, and were larger than those estimated by the Cox regression. However, the increase in precision for the Cox regression resulted in relatively poor coverage probabilities due to its large bias. The MPL coverage probabilities were close to its nominal value of 95%. For the same event proportions, both methods improved in bias and coverage probability when a smaller proportion of left and interval censored times were observed. This is partly due to the decreased width of the intervals that is necessary to produce a smaller proportion of left and interval censored data for the same set of randomly generated $t_{i}$ under our data generation process. As the sample size increases, the coverage probabilities of the MPL method are maintained around the 0.95 level, however, are reduced for the Cox with midpoint *t* method. In study 3 (Supplemental Table 1), we observe an improvement in the performance of the Cox regression with reduced bias and coverage probabilities close to the nominal level of 0.95. However, some parameters are still estimated poorly, particularly when estimating coefficient values for risk 1, with coverage probabilities decreased with increased sample size. For MPL, all coverage probabilities are close to the nominal 0.95 level.

**Figure 1.**
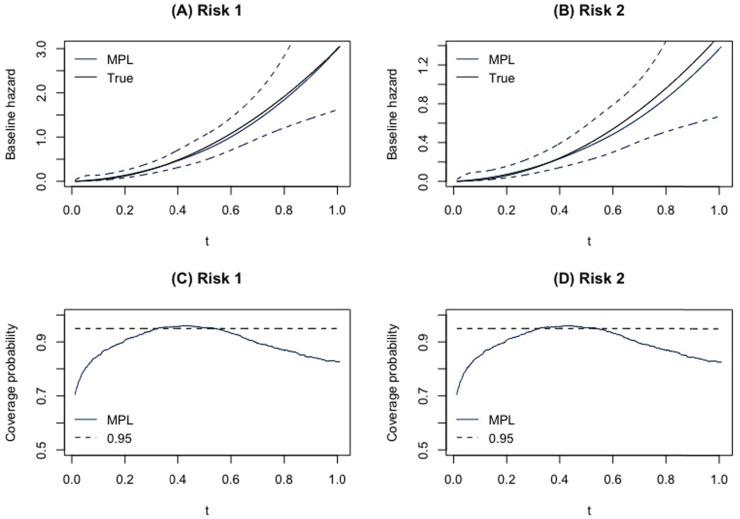
(A) and (B) The average baseline hazards for 1000 Monte Carlo runs, the true baseline hazards and the confidence bands for risks 1 and 2, respectively. (C) and (D) The coverage of the baseline hazards for risks 1 and 2, respectively.

**Figure 2.**
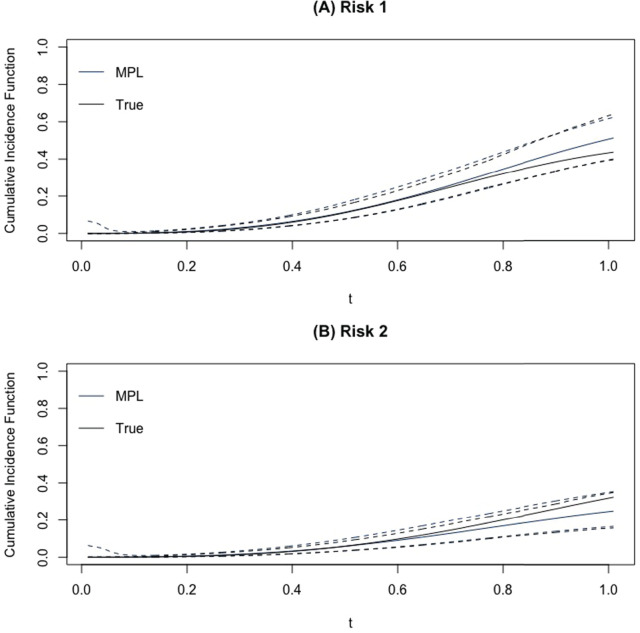
(A) and (B) The average of cumulative incidence functions (solid blue line) for 1000 Monte Carlo runs for scenario 1 with n=200 . Black lines represent the true cumulative incidence function (CIF) and confidence bands from the Monte Carlo runs are dashed lines.

**Figure 3.**
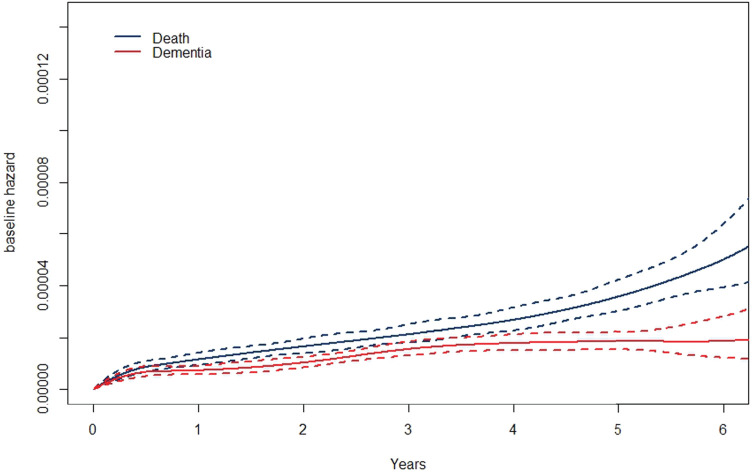
Baseline hazards for death (blue) and dementia (red) and their confidence bands based on the MPL in the ASPREE study. MPL: maximum penalized likelihood; ASPREE: Aspirin in Reducing Events in the Elderly.

**Figure 4.**
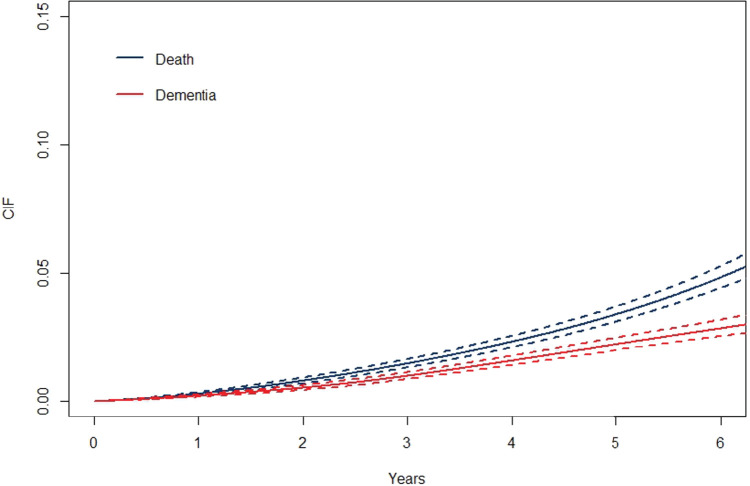
Cumulative incidence functions for death (blue) and dementia (red) and their confidence bands based on MPL in the ASPREE study. MPL: maximum penalized likelihood; ASPREE: Aspirin in Reducing Events in the Elderly.

Figure 1(A) and (B) shows the estimated average estimated baseline hazards for scenario 1 for the sample size of 200, and 47.5% right censoring proportion are estimated to be close to their true values for risks 1 and 2, respectively. The true baseline hazards for both risks are contained within the confidence bands. Figure 1(C) and (D) shows the coverage probabilities of the baseline hazards over t for the two risks. In general, the coverage probability is maintained close to the nominal 0.95 level and remains above 0.8 for the majority of the range. It is evident that the coverage probabilities are closer to the nominal 95% value when t is around (0.3,0.6) for both risks, and the coverage probability decreases as t approaches 0 and 1. This phenomenon occurs because most generated survival times are located closer to the middle of (0,1) , and survival times become scarce when t is close to 0 or 1. Figures 2 to 4(A) and (B) show the baseline cumulative incidence function graphs for risks 1 and 2, respectively. The true value is close to the average of the estimated CIF’s for both risks and within the estimated confidence bands.

The simulations were conducted on a 2021 MacBook Pro with an Apple M1 Pro chip and 16 GB RAM. For study 1, with approximate right censoring proportions of 47.5% and interval censoring (including left censoring) proportions of 47.5% and n=200 , these simulated data were estimated between 1 and 60 s (average 17 s). For the n=1000 sample size, the data were estimated between 23 and 777 s (average 165 s).

### 5.2. Application to dementia dataset

This study was initiated to explore the time to dementia in elderly patients while accounting for the competing risk of death and baseline covariates such as age, gender, education level, country of birth, and living arrangements (live alone or with others). The data was sourced from the “ASPirin in Reducing Events in the Elderly” (ASPREE) trial, a clinical trial conducted in Australia and the United States to determine whether daily use of 100 mg of enteric-coated aspirin would prolong the healthy lifespan of older adults.^21^ The trial included 19,114 relatively healthy older individuals, with 9525 randomized into the intervention group (aspirin) and 9589 into the control group (placebo). The participants were followed up every year for a maximum of 7 years. We selected dementia as an appropriate interval censored outcome since its diagnosis typically occurs over an extended period and in this trial was assessed every 2 years.

There were 963 (5%) deaths (event data) and 575 (3%) dementia cases (interval censored), and the remaining 17,576 (92%) were right censored. The hazard ratios estimated under both methods are largely in agreement for both outcomes. The standard errors in all parameters tend to be slightly larger for the MPL as observed in the simulation study. The *p*-values based on a cut-off of <0.05 would also agree in terms of significance testing. We suspect the large proportion of right censored observations in this dataset resulted in the similarity of these results. For death, the MPL results show that older age (HR 1.12, p<0.001 ), male (HR 1.73, p<0.001 ), less educated (HR 1.23, p=0.009 ), country of birth in the US (HR 1.29, p=0.026 ) and those that live with others (HR 1.27, p=0.004 ) were more likely to die (Table 3). For dementia, older age (HR 1.14, p<0.001 ), male (HR 1.41, p<0.001 ) and country of birth in the US (HR 1.39, p=0.019 ) were more likely to be identified with dementia.

**Table 3.** MPL and Cox (midpoint *t*) regression parameter estimates for death and dementia in the ASPREE trial.

	MPL			Cox (midpoint *t*)		
Death outcome	HR	SE (coef)	*p*-value	HR	SE (coef)	*p*-value
Age	1.123	0.007	<0.001	1.119	0.006	<0.001
Male versus female	1.73	0.078	<0.001	1.851	0.068	<0.0001
<13 years versus 13+ year education	1.233	0.08	0.009	1.215	0.069	0.005
COB: US versus Australia	1.287	0.113	0.026	1.253	0.098	0.0212
Living situation: with others versus alone	1.268	0.081	0.004	1.297	0.07	<0.001
Dementia outcome						
Age	1.138	0.009	<0.001	1.129	0.008	<0.001
Male versus female	1.41	0.098	<0.001	1.339	0.087	<0.001
<13 years versus 13+ year education	1.11	0.1	0.2966	1.122	0.089	0.1952
COB: US versus Australia	1.382	0.138	0.0187	1.445	0.12	0.0022
Living situation: with others versus alone	1.202	0.102	0.071	1.167	0.09	0.0864

*Note:* Living situation, “with others” includes living at home with family, in a residential home (with or withour supervised care). “Alone” includes living alone at home. MPL:maximum penalized likelihood. Cox (midpoint *t*) refers to the midpoint of interval censored times being treated as event times in a Cox regression; ASPREE: Aspirin in Reducing Events in the Elderly.

**Figure 5.**
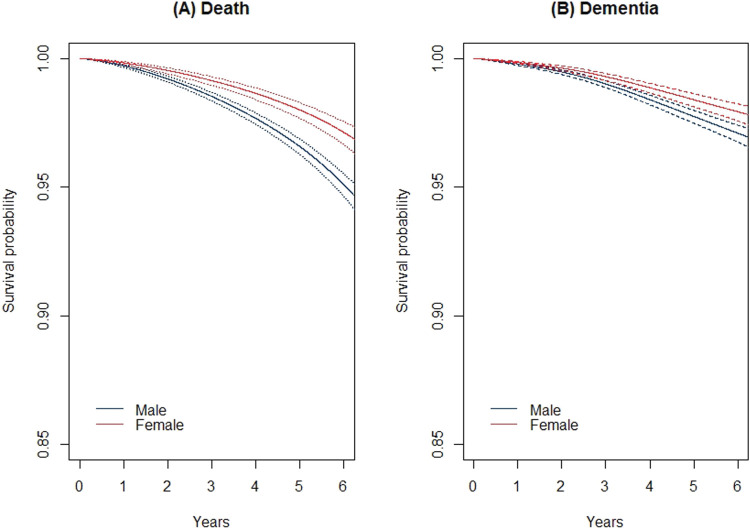
Survival curves for death (a) and dementia (b) by sex and their confidence bands based on MPL in the ASPREE study. MPL: maximum penalized likelihood; ASPREE: Aspirin in Reducing Events in the Elderly.

Figure 3 shows the baseline hazards of death and dementia. Based on this, we can see the risk of death is greater than dementia throughout the study period. Figure 4 shows the baseline cumulative incidence functions for the two outcomes. We can see that the incidence is higher in death than in dementia, however, based on their confidence bands is not significantly different. Figure 5 shows the survival curves of death (Figure 5(A)) and dementia (Figure 5(B)) by sex. In both outcomes, males’ survival curves are significantly lower than that of females.

## 6. Conclusions

When compared with the method of sieves for semi-parametric models, an advantage of penalty is that it makes the optimal number of basis functions and knots less important since the smoothing parameter can correct under or over smoothing. The MPL method is implemented in R and currently available on Github at https://github.com/josephdescallar/phcshMPL.

In this article, we also develop asymptotic properties of the constrained MPL estimates. Any semi-parametric PH regression model contains a baseline hazard as the non-parametric component and regression coefficients as the parameters of interest.

For inference of semi-parametric proportional hazard (PH) regression models, one unfortunate weakness of Cox’s partial likelihood or its modifications is that they cannot provide accurate risk predictions for individuals, which can badly limit applications of PH models. For likelihood based methods, although they are able to supply accurate individual predictions as they estimate baseline hazard and regression coefficients together, asymptotic results are more difficult to derive due to possibilities of active constraints.

## Supplemental Material

sj-pdf-1-smm-10.1177_09622802241262526 - Supplemental material for Cause-specific hazard Cox models with partly interval censoring – Penalized likelihood estimation using Gaussian quadrature Supplemental material, sj-pdf-1-smm-10.1177_09622802241262526 for Cause-specific hazard Cox models with partly interval censoring – Penalized likelihood estimation using Gaussian quadrature by Joseph Descallar, Jun Ma, Houying Zhu, Stephane Heritier and Rory Wolfe in Statistical Methods in Medical Research
