# Supplementary material for: Cause-specific hazard Cox models with partly interval censoring – Penalized likelihood estimation using Gaussian quadrature
Source: Stat Methods Med Res. 2024 Jul 25;33(9):1531–45. doi: 10.1177/09622802241262526 (PMC11523552; doi:10.1177/09622802241262526)
Supplement: sj-pdf-1-smm-10.1177_09622802241262526 - Supplemental material for Cause-specific hazard Cox models with partly interval censoring – Penalized likelihood estimation using Gaussian quadrature [file sj-pdf-1-smm-10.1177_09622802241262526.pdf]

Online Materials for "Cause-specific hazard Cox models with partly interval censoring – penalized likelihood estimation using Gaussian quadrature"

Joseph Descallar[1, 2, 3], Jun Ma *[1], Houying Zhu[1], Stephane Heritier[4], and Rory Wolfe[4]

[1]School of Mathematical and Physical Sciences, Macquarie University, Australia
[2]Ingham Institute for Applied Medical Research, Sydney, Australia
[3]South West Sydney Clinical Campuses, School of Medicine, UNSW, Sydney Australia
[4]School of Public Health and Preventive Medicine, Monash University, Australia

Large sample properties

Let n_r be the number of events from risk r and assume, for any r, $n_r \to \infty$ when $n \to \infty$.

We allow $m_r \to \infty$ as $n_r \to \infty$ but at a slower rate so that $m_r/n_r \to 0$ as $n_r \to \infty$.

For following assumptions are necessitated for these results in Theorems 1 & 2.

Assumptions

A1. The triplets $(T_i, D_i, \mathbf{X}_i^T)$, $1 \leq i \leq n$, are independently and identically distributed, and the distribution of $\mathbf{X}_i$ is independent of $\boldsymbol{\beta}$ and $h_{r0}(t)$ $(r = 1, \ldots, g)$.

A2. The objective function $\Phi(\boldsymbol{\beta}, \boldsymbol{\theta})$ is bounded.

A3. Each censoring time, including left, right, and interval censoring time, is independent of the corresponding event time, conditional on the covariates (see, for example, Sun[1] for the definition of independent interval censoring).

A4. Let Ω be the domain for vector $\boldsymbol{\eta}$. Corresponding to the true parameter value $\boldsymbol{\eta}_0 = (\boldsymbol{\beta}_0^\top, \boldsymbol{\theta}_0^\top)^\top \in \Omega$, the estimating equations $E_{\boldsymbol{\eta}_0}[n^{-1}\mathbf{U}^\top \partial\Phi(\boldsymbol{\eta})/\partial\boldsymbol{\eta}] = \mathbf{0}$ has a unique solution at $\widehat{\boldsymbol{\eta}} \in \Omega$, which is not necessarily $\boldsymbol{\eta}_0$, where $\Phi(\boldsymbol{\eta})$ is the penalized log-likelihood.

A5. The objective function $\Phi(\boldsymbol{\eta})$ is bounded for $\boldsymbol{\eta} \in \Omega$.

*To whom correspondence should be addressed: jun.ma@mq.edu.au

A6. $\Phi(\boldsymbol{\eta})$ is continuous over Ω and is twice differentiable in a neighbourhood of $\widehat{\boldsymbol{\eta}}$. Also, the matrices

$$\mathbf{G}(\widehat{\boldsymbol{\eta}}) = -\partial^2 l(\widehat{\boldsymbol{\eta}})/\partial\boldsymbol{\eta}\partial\boldsymbol{\eta}^\top \quad \text{and} \quad \mathbf{F}(\widehat{\boldsymbol{\eta}}) = -\partial^2 \Phi(\widehat{\boldsymbol{\eta}})/\partial\boldsymbol{\eta}\partial\boldsymbol{\eta}^\top.$$

Assume $\mathbf{U}^\top\mathbf{F}(\eta)\mathbf{U}$ is non-singular in a neighbourhood of $\widehat{\boldsymbol{\eta}}$.

A7. For each r, $J_r(\boldsymbol{\eta})$ is continuous and bounded over Ω, and $\partial J_r(\boldsymbol{\eta})/\partial\boldsymbol{\eta}$ and $\partial^2 J_r(\boldsymbol{\eta})/\partial\boldsymbol{\eta}\partial\boldsymbol{\eta}^\top$ exist for all $\boldsymbol{\eta} \in \Omega$. Moreover, $\partial^2 J_r(\boldsymbol{\eta})/\partial\boldsymbol{\eta}\partial\boldsymbol{\eta}^\top$ is bounded in a neighbourhood of $\widehat{\boldsymbol{\eta}}$.

Score vector and Hessian matrix elements

The components of the score vector of the penalised likelihood are as follows. Let x_{ij} be element j of vector x_i for $j = 1, ..., p$. Then the first derivative of $\Phi(\boldsymbol{\eta})$ with respect to β_{rj} is

$$\begin{aligned}\frac{\partial\Phi(\boldsymbol{\eta})}{\beta_{rj}} =& \sum_{r=1}^{n} x_{ij}\Bigg[\kappa_{ir}\delta_i - (\delta_i(1-\kappa_{i0}) + \kappa_{i0}) H_r(t_i) + \delta_i^L \sum_{q=1}^{g} \kappa_{iq}\frac{A_{qri}(t_i)}{F_q(t_i)} \\ &+ \delta_i^I \sum_{q=1}^{g} \kappa_{iq}\frac{A_{qri}\left(t_i^R\right) - A_{qri}\left(t_i^L\right)}{F_q\left(t_i^R\right) - F_q\left(t_i^L\right)}\Bigg]\end{aligned}$$

where

$$A_{qri}(t_i) = \int_0^{t_i} h_q(w) S(w) \left(1_{\{q=r\}} - H_r(w)\right) dw$$

The first derivative of $\Phi(\boldsymbol{\eta})$ with respect to θ_{ru} is

$$\begin{aligned}\frac{\partial\Phi(\boldsymbol{\eta})}{\partial\theta_{ru}} =& \sum_{i=1}^{n}\Bigg[\kappa_{ir}\delta_i\frac{\psi_{ru}(t_i)}{h_{0r}(t_i)} - (\delta_i(1-\kappa_{i0}) + \kappa_{i0})\Psi_{ru}(t_i) e^{\boldsymbol{X}_i\boldsymbol{\beta}_r} \\ &+ \delta_i^L \sum_{q=1}^{g} \kappa_{iq}\frac{B_{qrui1}(t_i) - B_{qrui2}(t_i)}{F_q(t_i)} e^{\boldsymbol{X}_i\boldsymbol{\beta}_r} \\ &+ \delta_i^I \sum_{q=1}^{g} \kappa_{iq}\frac{B_{qrui1}\left(t_i^R\right) - B_{qrui2}\left(t_i^R\right) - B_{qrui1}\left(t_i^L\right) + B_{qrui2}\left(t_i^L\right)}{F_q\left(t_i^R\right) - F_q(t_i)} e^{\boldsymbol{X}_i\boldsymbol{\beta_r}}\Bigg]\end{aligned}$$

where

$$B_{qrui1}(t_i) = \int_0^{t_i} 1_{\{q=r\}}\psi_{ru}(w) S(w) dw$$

and

$$B_{qrui2}(t_i) = \int_0^{t_i} h_q(w)\Psi_{ru}(w) S(w) dw$$

The components of the Hessian matrix are as follows.

$$\begin{aligned}\frac{\partial^2\Phi(\boldsymbol{\eta})}{\partial\beta_{rj}\partial\beta_{ck}} =&\sum_{i=1}^{n} x_{ij}x_{ik}\Bigg[(-1)_{\{r=c\}}\left(\delta_i(1-\kappa_{i0})+\kappa_{i0}\right)H_c(t_i)\\ &+\delta_i^L\sum_{q=1}^{g}\kappa_{iq}\frac{F_q\left(t_i\right)A_{qrjAck}\left(t_i\right)-A_{qrj}(t_i)A_{qck}(t_i)}{F_q\left(t_i\right)^2}\\ &+\delta_i^I\sum_{q=1}^{g}\kappa_{iq}\frac{\left(F_q\left(t_i^R\right)-F_q\left(t_i^L\right)\right)\left(A_{qrjAck}\left(t_i^R\right)-A_{qrjAck}\left(t_i^L\right)\right)}{\left(F_q\left(t_i^R\right)-F_q\left(t_i^L\right)\right)^2}\\ &-\frac{\left(A_{qrj}\left(t_i^R\right)-A_{qrj}\left(t_i^L\right)\right)\left(A_{qck}\left(t_i^R\right)-A_{qck}\left(t_i^L\right)\right)}{\left(F_q\left(t_i^R\right)-F_q\left(t_i^L\right)\right)^2}\Bigg]\end{aligned}$$

where

$$\begin{aligned}\frac{\partial A_{qrj}\left(t_i\right)}{\partial\beta_{ck}} =&x_{ik}A_{qrjAck}\left(t_i\right)\\ =&x_{ik}\int_0^{t_i}h_q\left(w\right)S\left(w\right)\Big(H_r\left(w\right)H_c\left(w\right)+(1)_{\{r=c,q=r\}}\\ &-\{(3)_{\{r=c,q=r\}},(1)_{\{r=c,q\neq r|r\neq c,q=c\}}\}H_r\left(w\right)-(1)_{\{q=r\}}H_c(w)\Big)dw\end{aligned}$$

$$\begin{aligned}\frac{\partial^2\Phi(\boldsymbol{\eta})}{\partial\beta_{rj}\partial\theta_{cu}} =&\sum_{i=1}^{n} x_{ij}e^{\boldsymbol{X_i\beta_c}}\Bigg[(-1)_{\{r=c\}}\left(\delta_i(1-\kappa_{i0})+\kappa_{i0}\right)\Psi_{cu}(t_i)\\ &+\delta_i^L\sum_{q=1}^{g}\kappa_{iq}\frac{F_q\left(t_i\right)A_{qrjBcu}\left(t_i\right)-A_{qrj}\left(t_i\right)\left(B_{qcu1}(t_i)-B_{qcu2}(t_i)\right)}{F_q\left(t_i\right)^2}\\ &+\delta_i^I\sum_{q=1}^{g}\kappa_{iq}\frac{\left(F_q\left(t_i^R\right)-F_q\left(t_i^L\right)\right)\left(A_{qrjBcu}\left(t_i^R\right)-A_{qrjBcu}\left(t_i^L\right)\right)}{\left(F_q\left(t_i^R\right)-F_q^L\left(t_i^L\right)\right)^2}\\ &-\frac{\left(A_{qrj}\left(t_i^R\right)-A_{qrj}\left(t_i^L\right)\right)\left(B_{qcu1}\left(t_i^R\right)-B_{qcu2}\left(t_i^R\right)-B_{qcu1}\left(t_i^L\right)+B_{qcu2}\left(t_i^L\right)\right)}{\left(F_q\left(t_i^R\right)-F_q^L\left(t_i^L\right)\right)^2}\Bigg]\end{aligned}$$

where

$$\begin{aligned}\frac{\partial A_{qrj}\left(t_i\right)}{\partial\theta_{cu}} =&e^{\boldsymbol{X_i\beta_c}}A_{qrjBcu}(t_i)\\ =&e^{\boldsymbol{X_i\beta_c}}\int_0^{t_i}(1)_{\{r=c,q=r\}}S\left(w\right)\psi_{ru}\left(w\right)+h_q\left(w\right)S\left(w\right)H_r\left(w\right)\Psi_{tu}\left(w\right)\\ &-\{(2)_{\{r=c,q=r\}},(1)_{\{r=c,q\neq r|r\neq c,q=r\}}\}h_q\left(w\right)S\left(w\right)\Psi_{tu}\left(w\right)\\ &-(1)_{\{r\neq c,q=c\}}S\left(w\right)H_r\left(w\right)\psi_{cu}\left(w\right)dw\end{aligned}$$

$$
\begin{aligned}
\frac{\partial^2\Phi(\boldsymbol{\eta})}{\partial\theta_{ru}\partial\theta_{cz}} &= \sum_{i=1}^{n}\Bigg[(-1)_{\{r=c\}}\kappa_{ir}\delta_i\frac{\psi_{ru}(t_i)\psi_{cz}(t_i)}{(h_{0r}(t_i))^2} \\
&+ \delta_i^L\sum_{q=1}^{g}\kappa_{iq}\Bigg(\frac{F_q\left(t_i\right)\left(B_{qru1Bcz}\left(t_i\right)-B_{qru2Bcz}\left(t_i\right)\right)}{F_q\left(t_i\right)^2} \\
&- \frac{\left(B_{qru1}(t_i)-B_{qru2}(t_i)\right)\left(B_{qcz1}(t_i)-B_{qcz2}(t_i)\right)}{F_q\left(t_i\right)^2}\Bigg)e^{\boldsymbol{X_i\beta_r}}e^{\boldsymbol{X_i\beta_c}} \\
&+ \delta_i^I\sum_{q=1}^{g}\kappa_{iq}\Bigg(\frac{\left(F_q\left(t_i^R\right)-F_q^L\left(t_i^L\right)\right)}{\left(F_q\left(t_i^R\right)-F_q^L\left(t_i^L\right)\right)^2} \\
&\times \frac{\left(F_q\left(t_i^R\right)-F_q^L\left(t_i^L\right)\right)\left(B_{qrui1Bcz}\left(t_i^R\right)-B_{qrui2Bcz}\left(t_i^R\right)-B_{qrui1Bcz}\left(t_i^L\right)+B_{qrui2Bcz}\left(t_i^L\right)\right)}{\left(F_q\left(t_i^R\right)-F_q^L\left(t_i^L\right)\right)^2} \\
&- \frac{\left(B_{qrui1}\left(t_i^R\right)-B_{qrui2}\left(t_i^R\right)-B_{qrui1}\left(t_i^L\right)+B_{qrui2}\left(t_i^L\right)\right)}{\left(F_q\left(t_i^R\right)-F_q^L\left(t_i^L\right)\right)^2} \\
&\times \frac{\left(B_{qcz1}\left(t_i^R\right)-B_{qcz2}\left(t_i^R\right)-B_{qcz1}\left(t_i^L\right)+B_{qcz2}\left(t_i^L\right)\right)}{\left(F_q\left(t_i^R\right)-F_q^L\left(t_i^L\right)\right)^2}\Bigg)e^{\boldsymbol{X_i\beta_r}}e^{\boldsymbol{X_i\beta_c}}
\end{aligned}
$$

where

$$\frac{\partial B_{qru1}\left(t_i\right)}{\partial\theta_{cz}} = e^{\boldsymbol{X_i\beta_c}}B_{qru1Bcz}(t_i) = e^{\boldsymbol{X_i\beta_c}}\int_0^{t_i} -(1)_{\{q=r\}}S\left(w\right)\psi_{ru}\left(w\right)\Psi_{cz}\left(w\right)dw$$

and

$$\frac{\partial B_{qru2}\left(t_i\right)}{\partial\theta_{cz}} = e^{\boldsymbol{X_i\beta_c}}B_{qru2Bcz}(t_i) = e^{\boldsymbol{X_i\beta_c}}\int_0^{t_i}(1)_{\{q=c\}}S\left(w\right)\Psi_{ru}\left(w\right)\psi_{cz}\left(w\right)-h_q\left(w\right)S\left(w\right)\Psi_{ru}\left(w\right)\Psi_{cz}\left(w\right)dw$$

Simulation 3

Table 1: Cox (midpoint t) and MPL regression parameter estimation for study 3

				$\beta_{11} = -1$		$\beta_{12} = 0.5$		$\beta_{21} = 1$		$\beta_{22} = -0.5$	
n	int cens (%)	right cens (%)		Cox	MPL	Cox	MPL	Cox	MPL	Cox	MPL
200	47.5	47.5	bias	-0.068	0.024	0.034	-0.047	-0.010	-0.068	0.012	-0.027
			std asymp	0.138	0.160	0.243	0.258	0.220	0.234	0.392	0.397
			std mc	0.141	0.161	0.260	0.282	0.223	0.245	0.403	0.408
			cov prob	0.917	0.953	0.928	0.932	0.944	0.940	0.952	0.951
200	75	20	bias	-0.260	0.007	0.125	-0.045	0.044	-0.064	-0.022	-0.030
			std asymp	0.110	0.165	0.197	0.258	0.170	0.221	0.299	0.341
			std mc	0.121	0.166	0.210	0.264	0.185	0.229	0.305	0.342
			cov prob	0.337	0.955	0.888	0.944	0.921	0.944	0.952	0.949
1 000	47.5	47.5	bias	-0.273	0.022	0.136	-0.033	0.074	-0.015	-0.034	-0.015
			std asymp	0.048	0.076	0.087	0.114	0.072	0.099	0.130	0.151
			std mc	0.053	0.079	0.091	0.120	0.076	0.104	0.126	0.149
			cov prob	0.001	0.938	0.652	0.930	0.787	0.944	0.947	0.958
1 000	75	20	bias	-0.084	0.021	0.041	-0.028	0.017	-0.020	-0.013	-0.020
			std asymp	0.060	0.070	0.106	0.113	0.093	0.100	0.168	0.170
			std mc	0.062	0.073	0.113	0.123	0.093	0.102	0.164	0.170
			cov prob	0.678	0.933	0.914	0.924	0.944	0.942	0.951	0.948

Notes: MPL = Maximum penalized likelihood. Cox (Midpoint t) refers to the midpoint of interval censored times being treated as event times in a Cox regression.

References

[1] Sun J. *The statistical analysis of interval-censored failure time data*, New York: Springer, 2006.
